# Mannanoligosaccharides as a Carbon Source in Biofloc Boost Dietary Plant Protein and Water Quality, Growth, Immunity and *Aeromonas hydrophila* Resistance in Nile Tilapia (*Oreochromis niloticus*)

**DOI:** 10.3390/ani10101724

**Published:** 2020-09-23

**Authors:** Asmaa T.Y. Kishawy, Alaa H. Sewid, Hend S. Nada, Mohamed A. Kamel, Shefaa A.M. El-Mandrawy, Taghrid M.N. Abdelhakim, Abd Elhakeem I. El-Murr, Nihal El Nahhas, Wael N. Hozzein, Doaa Ibrahim

**Affiliations:** 1 Department of Nutrition and Clinical Nutrition, Faculty of Veterinary Medicine, Zagazig University, Al Sharqia Governorate 44519, Egypt; 2 Departments of Microbiology, Faculty of Veterinary Medicine, Zagazig University, Al Sharqia Governorate 44519, Egypt; veterinarian.alaa.sweed@gmail.com (A.H.S.); hend.saeed@hotmail.com (H.S.N.); 3 Department of Biomedical and Diagnostic Sciences, University of Tennessee, Knoxville, TN 37996, USA; 4 Department of Pharmacology, Faculty of Veterinary Medicine, Zagazig University, Al Sharqia Governorate 44519, Egypt; Kamelma78@gmail.com; 5 Department of Clinical Pathology, Faculty of Veterinary Medicine, Zagazig University, Zagazig 44519, Egypt; shifo_vet@yahoo.com; 6 Department of Fish Health and Management, Central Laboratory for Aquaculture Research, Agriculture Research Center, Abo Hammad 44519, Egypt; jodymt333@gmail.com; 7 Department of Fish Diseases and Management, Faculty of Veterinary Medicine, Zagazig University, Al Sharqia Governorate 44519, Egypt; Hakimelmor@zu.edu.eg; 8 Department of Botany and Microbiology, Faculty of Science, Alexandria University, Moharram baik, Alexandria 21515, Egypt; nihal.elnahhas@alexu.edu.eg; 9 Bioproducts Research Chair, Zoology Department, College of Science, King Saud University, Riyadh 11451, Saudi Arabia; whozzein@ksu.edu.sa; 10 Botany and Microbiology Department, Faculty of Science, Beni-Suef University, Beni-Suef 62511, Egypt

**Keywords:** biofloc, Nile tilapia, carbon sources, plant protein, fish protein, mannanoligosaccharides, *Aeromonas hydrophila*, virulence gene

## Abstract

**Simple Summary:**

Biofloc technology (BFT), offers some potential advantages for improvements in water quality and growth of farmed fish reared in recirculation systems. One practical disadvantage of implementing a BFT system to culture fish is the need to add organic carbon to maintain a C:N ratio above 10. The present study evaluated the effect of using mannan oligosaccharides as a carbon source in a biofloc system with cultivated tilapia. MOS resulted in increased lactic acid bacterial count in the water and the intestinal tract, modulated immune response and resistance against Aeromonus hydrophila and improved the survival and growth of reared Nile tilapia (*Oreochromis niloticus* L.)

**Abstract:**

The aim of the present study was to evaluate mannan oligosaccharides (MOS) or glycerol (GLY) as a carbon source on biofloc systems of Nile tilapia (*O. niloticus)* juveniles. Fish (*n* = 750) were reared in open flow (Controls) or biofloc systems (B-GLY and B-MOS) fed with a plant or fish protein source over a period of twelve weeks. Total ammonia nitrogen and nitrate decreased in the biofloc groups, while biofloc volume increased in B-MOS. Compared to the controls, B-MOS and B-GLY exhibited higher weight gain and improved feed conversion, irrespectively of the diet. Serum level of C-reactive protein was reduced, while IgM and lysozyme activity was higher in the B-MOS fish, compared to other groups. Intestinal *Bacillus* spp. count was increased, whereas *Vibrio, Aeromonas* and *Pseudomonas* spp. counts decreased in B-MOS reared groups, compared to the other groups. The proinflammatory cytokine (IL-8 and IFN-γ) transcript expression was upregulated in B-MOS more than B-GLY reared groups. Compared to the controls, the virulence of *Aeromonas hydrophila* was decreased in the B-MOS and B-GLY groups. The results indicate several benefits of using MOS as a carbon source in a biofloc Nile tilapia system; a cost benefit analysis is required to assess the economic viability of this.

## 1. Introduction

The pollution of culture water in the semi-intensive and intensive systems for fish culture is due to the accumulation of fish waste and uneaten feed, as about 26% nitrogen and 30% phosphorus from feed are utilized by fish, the remainder causing an increase in bond nitrogen and ammonia load [1]. Continuous and partial water exchange is necessary, especially in intensive fish culture, to maintain good water quality, which may cost about 10% of total production cost [2]. Biofloc technology (BFT) is a recent technique developed to maintain water quality through up-taking water nitrogen and converting it to microbial protein [3]. Bioflocs comprise a mixture of heterogeneous floc-former microorganisms such as bacteria, microalgae, protozoa, phytoplankton, rotifers, annelids, nematodes, copepods, cations, colloids, organic polymers, uneaten feed and dead cells [4]. All floc formers aggregate with each other, forming a mass that fish can feed on and supporting nutrient recycling and improving fish growth performance [5,6]. The protein in the commercial fish diet can be decreased from 35% to 24% under the biofloc rearing system due to the presence of microbial protein that compensates for the low amount of dietary protein [7].

One of the primary requisites of biofloc formation is to maintain the carbon to nitrogen ratio in the culture system. As for the development of biofloc, the carbon/nitrogen ratio should range from 10:1 to 20:1, and the carbon sources may be varied [8]. Fish reared in the biofloc system have a higher improved immune response and disease resistance [9,10].

Glycerol considered an organic source of carbon which is easily soluble in water [11]. Glycerol is used as an economic carbon source in a biofloc system of fish rearing to neutralize ammonia waste in water [12]; also, Ekasari et al. [13] reported that the use of glycerol increased long chain fatty acid amount in biofloc; moreover, the increased level of glycerol to reach 1:15 C:N produced the best balance between water quality and nutritive value of biofloc [12]. The addition of complex carbohydrate as a carbon source in a biofloc system can enhance the nutritive value of bioflocs, which have been suggested as a contributor to the better growth of fish compared to glycerol [12].

Mannan oligosaccharides (MOS) are a prebiotic that is indigestible by mammalian and fish enzymes but digested by microbial enzymes [14]. Mannan oligosaccharides are reported to improve growth performance, feed conversion, immune response such as lysozymes activity and total IgM, and increase intestinal lactic acid bacteria in common carp [15]. *Bacillus subtilis* bacteria have β-mannanase enzymes that can hydrolyze mannan oligosaccharides and use them for its proliferation [16] and production of their metabolites, which are mainly lactic acid and acetic acid [17]. The proliferation of lactic acid bacteria in water or the intestine suppresses the colonization of Gram-negative pathogenic bacteria [18]; moreover, pathogenic bacteria proliferation can be eliminated by MOS itself, as it is capable of attaching to glycoprotein receptors of pathogens, preventing their attachment and colonization [19]. The combination of both MOS and lactic acid bacteria may have a beneficial effect on fish by promoting performance, immunity and disease resistance [20]. Determining the best carbon source to be used in the biofloc system with growth and immune-modulating effects, together with benefiting the quality and quantity of biofloc, may add other advantages from using biofloc technology in the Nile tilapia *Oreochromis niloticus* culture system. As fish meal in fish feed is suffering from low supply, massive demand and higher costs, using less by adopting an alternative plant protein with natural food from biofloc will optimize the production of Nile tilapia. The immunomodulatory effect of MOS has been observed in previous studies [15,21]. To the best of our knowledge, this is the first study to address the addition of MOS as a prebiotic and carbon source in a biofloc system.

The objective of this study is to determine the effect of the inclusion of glycerol or MOS as a carbon source in biofloc system water with two dietary protein sources on the growth performance, modulation of intestinal and water microbiota, immune response and disease resistance to *Aeromonas hydrophila* in Nile tilapia fingerlings.

## 2. Material and Methods

### 2.1. Ethical Statement

The experimental protocol and ethics were performed following the recommendations of the local experimental fish care committee of the Faculty of Veterinary Medicine, Zagazig University, and was accepted by the faculty ethics committee (Approval No. ZU-IACUC/5/M/39/2019)

### 2.2. Experimental Design and Biofolc Preparation

Nile tilapia fingerlings (*Oreochromis niloticus*) were obtained alive and observed to be apparently healthy from a private farm in Kafr El-Sheikh Governorate, Egypt. On arrival to the lab, fish were acclimated to experimental conditions in fiberglass tanks for two weeks, during which fish were fed a basal diet. After that, Nile tilapia were randomly stocked in 30 glass aquaria (120 × 80 × 60 cm) as experimental units supplied with air compressors with two ceramic air diffuser discs (13 cm diameter × 20 mm length) per tank.

#### 2.2.1. Experimental Design

A total of 750 Nile tilapia with an initial experimental weight of 8.2 ± 0.2 g/fish were randomly divided into six treatments (five replicates per treatment; *n* = 25 fish/replicate) for 12 weeks. The experimental groups consisted of two control groups, in which fish were reared on clear water (without carbon sources and 25% daily water exchange) and fed on a plant or fish protein-based diet (C-plant protein and C-fish protein), respectively. The other four groups were reared on a biofloc system as follows: third and fourth fish groups were reared on biofloc system based on glycerol (as a carbon source) and fed on plant protein or fish protein-based diet (B-GLY plant protein and B-GLY fish protein), respectively, while the fifth and six fish groups were reared on a biofloc system based on mannan oligosaccharides and fed on a plant protein or fish protein-based diet (B-MOS plant protein and B-MOS fish protein), respectively.

#### 2.2.2. Biofloc Preparation

Two carbon sources (glycerol and mannan oligosaccharides) were used to prepare biofloc water inoculants. Primarily, two polyethylene tanks of 20 L capacity (each tank was supplied with one air stone connected to the central compressor) were filled with screen filtered effluent water from a tilapia culture tank, and fresh dechlorinated water was added at a ratio of 1:10. Additionally, (NH_4_)_2_SO_4_, NH_4_Cl, Na_2_HPO_4_ and NaSiO_3_ were added to each tank at 10, 95.54, 94.7 and 1 mg/L, respectively, to simulate nitrogen loading in a biofloc system [13]. For the preparation of glycerol based biofloc water, 40 mg/L glycerol (39.19 % of C) (obtained from Sigma Aldrich Chemie, Merck KGaA, Darmstadt, Germany, with MDL number MFCD00004722) was added to one tank of the previously prepared mixture. For the preparation of MOS based biofloc water, 61.96 mg/L mannan oligosaccharides (MOS) (38.10 % of C) (from Sigma Aldrich Chemie, Merck KGaA, Darmstadt, Germany, MDL number MFCD00131574) was added to the other tank; the temperature was adjusted to 28 ± 2 °C with natural day light conditions. After 48 h, the floc volume was 5.3 mL/L; so, it was left for one week to increase floc volumes before the stocking of fish (volume of biofloc was 13.7 mL/L).

### 2.3. Experimental Setup and Diets Formulation

Nile tilapia fingerlings in the control groups were reared under a regular daily water exchange system. Meanwhile, the biofloc groups were reared under a static system without water exchange, where the previously prepared biofloc water was mixed with the experimental tank’s freshwater at the ratio of 1:100 (biofloc water: freshwater) [22]. In order to maintain fully developed biofloc or microbial community conditions, C/N ratio was maintained at 15:1 by the addition of daily carbon sources by dissolving the adjusted amount of each source (glycerol or MOS) in 50 mL water, which was then added to each corresponding tank. The amount of carbon added was calculated according to [23] as a demand carbon source = 1 g diet (28 g diet protein/100 g diet) (16 g diet nitrogen/100 g diet protein) (75 g waste nitrogen/100 g diet nitrogen) (15 g carbon/1 g waste nitrogen) (100 g carbon source /the amounts of carbon (g) in carbon source). 

Basal diets were formulated according to nutrient requirement tables of National Research Council (NRC) for Nile tilapia (*O. niloticus*) according to [24] Table 1. The ingredients of experimental diets were blended and homogenized using a mixture grinder (225 μm particle size for different feed ingredients), then oil was added and finally pelleted using a pelleting machine with 2 mL diameter; then, pellets were air dried for 24 h at 24 °C. The fish was fed at the rate of 3 % of live body weight daily and feeding was distributed about four times per day for 12 weeks. Chemical analysis (moisture, crude protein and ether extract, ash) of the feed ingredients was conducted according to the official methods of [25].

### 2.4. Water Quality Analysis

The temperature, dissolved oxygen using optical dissolved oxygen meter (YSI Pro ODO, Yellow Spring Instruments, Yellow Springs, OH, USA), pH using a pH meter (PSH-3C, INESA Instrument, Shanghai, China) and salinity were measured daily at 9.00 a.m. Total ammonia-nitrogen (TAN) using Nessler reagent spectrophotometric determination, nitrite-nitrogen using naphthyl ethylene diamine spectrophotometry, nitrate-nitrogen using ultraviolet spectrophotometer [26] and biofloc volume were measured once per week (the floc settlement in a Imhoff cone) by pouring one liter of tank water for 30 min [5] according to standard methods [27]. Dechlorinated tap water was used to change about 25% of the water every day in the control groups only.

### 2.5. Evaluation of Growth Performance

Average body weight (BW): all fish of each replicate were weighed individually at the beginning of the experiment to obtain initial body weight, and then, they were weighed at two-week intervals. Weight gain (WG), weight gain percentage (WG %), feed intake (FI), feed conversion ratio (FCR), protein efficiency ratio (PER), specific growth rate (SGR, %) and Fulton’s condition factor (K) were calculated accordingly during the experimental period as follows:

BW, g/fish = total weight of fish/number of fish per group.

WG, g/fish = final body weight–initial body weight.

WG% = ((final average body weight–initial average body weight)/initial average body weight) * 100 [28].

FI, g/fish = total feed consumed divided by the number of fish [29].

FCR = dry feed fed (g)/wet weight gain (g) [30].

PER = wet weight gain (g)/protein intake (g) [31].

SGR % = ((LN (final body weight) − LN (initial body weight))/time (days)) * 100 [32], where LN = natural log

K = (W/L^3^) * 100 [33], where W = total weight of fish (g), L = length of fish (cm) measured from the tip of the snout to the end of the middle caudal fin.

### 2.6. Digestibility

At the end of the experiment, ten fish of each replicate were kept in their rearing tanks for measuring the apparent digestion coefficient of different experimental diets using chromic oxide (Cr_2_O_3_) at a level of 5 g/kg diet as an external indicator. Feces of fish were collected for 14 days and kept at −20 °C till chemically analyzed for moisture, crude protein, ether extract, nitrogen-free extract and ash according to the official methods of [25]. Additionally, chromic oxide was analyzed in feces according to [34]; then, the apparent digestion coefficient (DC) of dry matter (DM), crude protein (CP), ether extract (EE), nitrogen-free extract (NFE) and ash was calculated according to [35]. 

### 2.7. Blood Sample Collection 

At the end of the experiment, ten fish per replicate were used for blood sample collection and anesthetized by dipping in 0.1 ppm suspension of Ethyl 3-aminobenzoate methanesulfonate (Sigma-Aldrich Chemie GmbH. Eschenstrasse. 5 D-82024 Taufkirchen, Germany: product No. E10521). Blood samples were collected from the caudal blood vessels at the caudal peduncle region using a 3 mL plastic syringe without anticoagulant and centrifuged at 3000 rpm for 15 min for separation of serum which was immediately used for the estimation of nonspecific immune parameters.

#### Immune Parameters Evaluation

Serum levels of total protein and albumin were measured spectrophotometrically by using specific kits according to [36,37]. Globulin levels were calculated by subtraction of the values of albumin from the values of total protein. The activity serum lysozyme was analyzed by lysoplate assay [38]. Serum Immunoglobulin M (IgM) was analyzed spectrophotometrically by Enzyme-Linked immunosorbent Assay (ELISA) kits (Cusabio Biotech Co. Ltd., Wuhan, China). Latex-enhanced nephelometry was used for detection of C-reactive protein (CRP), which depends on the reaction between a soluble analyte and the equivalent antigen or antibody attached to polystyrene particles according to [39].

### 2.8. Bacterial Counts in Water and Fish Intestine

Enumeration of the water bacterial count was carried out every two weeks (five samples/replicate), while fish intestine microbial population from intestinal content was carried out at the end of the experiment after blood sample collection (ten fish/replicate), used to collect intestinal content by cutting part of the intestine under sterile conditions and squeezing its content in a sterile Eppendorf tube, which was taken directly for analysis by the plate counting technique. In brief, a tenfold dilution for 1 mL intestinal content and water was prepared in sterilized saline. For each dilution, 0.1 mL was inoculated in three plates of Man–Rogosa–Sharpe agar plates (110660, Sigma-Aldrich, St. Louis, MO, USA) for lactic acid bacteria [40], cetrimide agar (17208, Sigma-Aldrich) for *Pseudomonas* spp. [41], tryptone soy agar plates (CM0131, Oxoid, Ottawa, ON, Canada) for *Aeromonas* spp. [42] and thiosulfate-citrate-biliary salts agar without salt (86348, Sigma-Aldrich, Germany) for nonhalophilic *Vibrio* spp. [43]. The morphology of the bacteria was observed by microscopic examination of the cells after purification of the bacterial colonies and consequent estimation of Gram type. The result was expressed as colony forming units (CFU) per mL of water or intestinal content.

### 2.9. Expression of Immune and Bacteria Related Genes

#### Immunity Genes Expression

At the end of the experimental period, spleen samples from the five fish groups were collected prechallenge in Eppendorf capped tubes and stored at −80 °C till further analysis. The total RNA was extracted from the spleen tissues using the QIAamp RNeasy Mini kit (Qiagen, Hilden, Germany, GmbH). Total RNA quantity and purity were determined using a NanoDrop ND-8000 spectrophotometer (Thermo Fisher Scientific, Waltham, MA, USA). The synthesis of cDNA was performed after RNA extraction as 25 µL reaction containing 12.5 µL of the 2 × QuantiTect SYBR Green PCR Master Mix (Qiagen, Hilden, Germany, GmbH), 0.25 µL RevertAid Reverse Transcriptase (200 U/µL) (Thermo Fisher), 0.5 µL of each primer of 20 pmol concentration, 8.25 µL RNase free water and 3 µL RNA template. The expression levels of immunity genes including IL-8 [44] and IFN-γ [45] gene expression were quantified using real-time PCR. The oligonucleotides sequence of used primer for β-actin, IL-8 and IFN-γ were supplied by Metabion (Planegg/Pteinkirchen, Germany) and listed in Table 2.). The relative gene expression of target genes was estimated by the comparative method (2^−ΔΔCt^). The β-actin [46] gene was selected as a control to normalize target gene expression levels [47]. 

### 2.10. Challenge Test with Aeromonas hydrophila

Five days after collection of the first samples, ten fish from each replicate were challenged, as they were injected intraperitoneal with 0.2 mL 24 h (3 × 10^8^ CFU) pathogenic *A. hydrophila* reference strains (ATCC 7966). Infected fish were examined for 7 d to record clinical symptoms, postmortem lesions and mortality rates. The average mortality among all replicates was used for calculating relative percent survival (RPS) after a challenge [48].

At seven days postchallenge, five fish/group were used for the collection of spleen tissues for quantization of DNA copies of the *A. hydrophila* strain and for examining the mRNA expression of its virulence gene (*A-hyd Aerolysin*).

#### 2.10.1. Bacterial Isolation

After the challenge, spleen samples were collected from challenged fish (five samples/replicate) for isolation of bacterial colonies and inoculated onto blood and tryptone soy agar plates (CM0271, Oxoid and CM0131, Oxoid, respectively) and incubated for at 28 °C for 48 h; then, the produced pure isolate from *Aeromonas* was stored in brain heart infusion broth medium (CM1135, Oxoid) with glycerol at −80°C until further analysis [49].

#### 2.10.2. Real-Time PCR Assay

Quantification of DNA copies of the *Aeromonas hydrophila* strain by real-time PCR: Total genomic DNA was extracted from the Nile tilapia spleen tissues postchallenge and bacterial isolates by using the Thermo Scientific GeneJET Genomic DNA purification kit according to the manufacturer’s protocol. The purity and concentration of gyrB gene in each sample were measured using a Nanodrop ND-1000 spectrophotometer (Implen NanoPhotometer™, GMBH, Munich, Germany). The reaction mixture contained extracted DNA, SYBR Green Master Mix (Qiagen, Chadstone, Australia) and forward and reverse primers of the specific target gene *A. hydrophila* (gyrB) [50] (Table 2).

The quantitative detection of *A. hydrophila* was performed by using the standard curve method in real-time PCR [51]. The standard curves were performed by using a number of copies of the *A. hydrophila* (gyrB) [50] plotted against the quantification cycle (Cq) taken from 10-fold serial dilutions of PCR products from a pure isolate of *A. hydrophila*. The quantitative PCR assays were conducted in a Rotor-Gene 6000 Series real-time PCR machine; the reaction was performed against a set of standards generated with the spiked samples of known concentrations. The *A. hydrophila* concentration in each DNA sample was calculated by interpolating the Ct values of DNA samples from spleen tissues into the generated standard calibration curves, and then, their log10 of the CFU numbers was estimated.

- Quantification of *Aeromonas hydrophila* strain by real-time PCR

The quantitative detection of *A. hydrophila* was performed by using extracted DNA, SYBR Green Master Mix (Qiagen, Chadstone, Australia) and forward and reverse primers of the specific target gene *A. hydrophila* (gyrB) [50] (Table 2). The Quantitative PCR assays were conducted in a Rotor-Gene 6000 Series real-time PCR machine; the reaction was run against a set of standards generated with the spiked samples of known concentrations. The *A. hydrophila* concentration in each DNA sample was calculated by interpolating the Ct values of DNA samples from spleen tissues into the generated standard calibration curves, and then, their log10 of the CFU numbers was estimated. The relative gene expression data were analyzed using the 2^−ΔΔCt^ method [52].

- Quantification of *Aeromonas hydrophila* virulence gene (A-hyd Aerolysin) by real-time PCR

Total RNA was extracted from fish spleen tissues using a QIAamp RNeasy Mini kit (Qiagen GmbH, Hilden, Germany). The concentration of the extracted RNA was assayed using a Spectrostar NanoDropTM 2000 spectrophotometer (Thermo Fisher Scientific Inc., Waltham, MA, USA). The RNA purity was then confirmed by the absorbance ratio (260 and 280 nm). cDNA was synthesized using kits of RevertAidTM H Minus, (Fermentas Life Science, Pittsburgh, PA, USA); then, 1 μL of this cDNA was mixed with 12.5 μL 2× SYBR^®^ Green PCR mix with ROX from BioRad, 5.5 μL RNase free water and 0.5 μL (10 pmol/μL) of each forward and reverse primer for *A-hyd Aerolysin* [53] were added Table 2. 16S rRNA gene was used as an internal housekeeping gene. The real-time PCR amplification and the analysis of relative gene expression were performed using the 2^−ΔΔCt^ method [52].

### 2.11. Statistical Analysis

The data were analyzed after confirming the homogeneity of variance among experimental groups by Levene’s test (the effects of the replicate were analyzed but not significant) by GLM (general linear model) two-way analysis of variance, using PASW statistics 18 (SPSS Inc., Chicago, IL, USA) to clarify the effects of biofloc carbon sources and dietary protein sources, and their interaction. 

Tukey’s test was used to separate the means when the treatment difference was significant at *p*-value < 0.05. Residual standard deviation (RSD) was calculated as the square root of the error mean square divided by the square root of the number of samples. 

## 3. Results

### 3.1. The Effect of Different Dietary Protein Sources and Carbon Sources on Water Quality Parameters of Nile Tilapia Rearing Water

During the experimental period, water quality parameters such as water temperature, dissolved oxygen and salinity showed little differences between all experimental groups (Table 3). Water pH tended to be more acidic in B-MOS groups than B-GLY groups than the control group. Total ammonia nitrogen (TAN), nitrite N and nitrate N were significantly lower (*p* < 0.05) in the biofloc system regardless of the diet protein source. The biofloc volume was significantly increased (*p* < 0.05) in biofloc reared groups compared to control groups. 

### 3.2. The Effect of Different Dietary Protein Sources and Carbon Source Nile Tilapia Growth Performance

The growth performance parameters (final body weight (BW) and weight gain (WG), feed conversion ratio (FCR), protein efficiency ratio (PER) and specific growth rate) of Nile tilapia reared in biofloc systems were significantly (*p* < 0.05) improved against those reared in normal conditions as the control (Table 4). The body gain increased significantly by 11.72% and 27.57% in glycerol-based biofloc groups and in MOS based biofloc groups, respectively, compared to the control groups. Using biofloc systems and dietary protein sources had no effect on feed intake through the experimental period. Rearing Nile tilapia in the biofloc system had a significantly (*p* < 0.05) improved feed conversion ratio under 0.9 compared to the control groups; however, the carbon source and protein source in the biofloc system did not have a significant effect on Nile tilapia FCR. The best specific growth rate (SGR %) was recorded (*p* < 0.05) in the B-MOS fed plant protein-based group. Moreover, PER of Nile tilapia reared in all biofloc groups recorded significantly (*p* < 0.05) higher PER than the control groups, while dietary protein sources had no significant effect on PER among experimental groups. Fulton’s condition factor (K) was significantly higher in all biofloc system groups—either MOS or glycerol fed with a plant protein-based or fish protein-based diet—than the control groups. Furthermore, the most prominent effect (*p* < 0.05) was in the B-MOS groups. 

### 3.3. The Effect of Different Dietary Protein Sources and Carbon Source on Nile Tilapia Nutrient Digestibility 

The application of the biofloc rearing system significantly affected the nutrient digestibility of Nile tilapia (Table 5). The digestibility of dry matter (DM), crude protein (CP) and ash exhibited significantly higher (*p* < 0.05) values in the fish-protein fed groups than groups fed on plant protein diets. Regarding the effect of carbon source, the improvement in DM, CP, ether extract (EE) and ash digestibility in the B-MOS reared groups revealed significant differences (*p* < 0.05) with the B-GLY reared groups and control groups. The digestibility of NFE was not affected by experimental treatments. Considering the interaction between carbon source and protein source, the digestibility of DM significantly increased (*p* < 0.05) in B-MOS reared fish (either fed on a fish or plant protein source) compared to the B-GLY groups (either fed on a fish or plant protein source). There was a significant interaction in CP digestibility between groups: The highest values were obtained in fish protein source groups reared on B-MOS followed by the group reared on B-GLY. CP digestibility in groups fed on a plant protein source was improved in B-MOS groups compared to B-GLY groups. Moreover, the fat digestibility was improved in all experimental groups reared in the biofloc system, with the highest values in the fish group reared on the B-MOS biofloc system with a fish protein source. The highest apparent ash digestibility was recorded for groups fed on fish protein source, especially those reared on the biofloc system (*p* < 0.05).

### 3.4. Effect of Different Dietary Protein Sources and Carbon Sources on Nile Tilapia Water Bacterial Count Every Two Weeks of 12-Week Experiment

**Figure 1:**
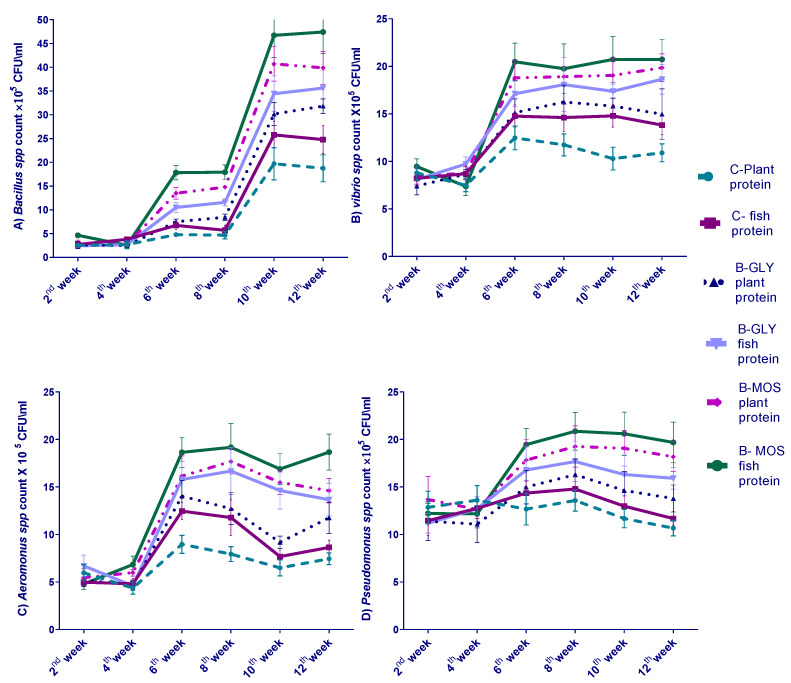
Water bacterial count for *Bacillus* spp., *Aeromonas* spp., *Vibrio* spp. and *Pseudomonas* spp. every two weeks of the 12-week experiment (cfu/mL) (data are expressed as mean ± SEM).

Isolation of bacteria from the water fish tanks was performed every two weeks throughout the 12-week experimental period. Control groups exhibited no significant change in *Bacillus* spp., *Vibrio* spp., *Aeromonas* spp. and *Pseudomonas* spp. during the experimental period (Figure 1). Our results indicated that all bacterial species counts significantly increased (*p* < 0.05) gradually throughout the experimental period in biofloc groups. The inclusion of MOS as a carbon source in the biofloc system mostly affected *Bacillus* spp., and it was the dominant bacteria group (46.71–47.41 × 10^5^ CFU mL^−1^) among measured bacterial populations in B-MOS groups.

*Vibrio* spp., *Aeromonas* spp. and *Pseudomonas* spp. counts were not significantly affected (*p* < 0.05) during 12 weeks of the experiment in control water, but they gradually increased in the water of biofloc groups either in B-MOS or B-GLY. 

### 3.5. The Effect of Different Dietary Protein Sources and Carbon Sources on Nile Tilapia Intestinal Bacterial Count 

The bacterial population isolated from fish intestinal content was not affected by dietary protein sources but was affcted by carbon sources in both biofloc systems (Table 6). The highest probiotic *Bacillus* count was observed in the B-MOS groups followed by the B-GLY groups when compared with the control groups. The counts of pathogenic bacteria such as *Vibrio* spp. and *Aeromonas* spp. were significantly decreased (*p* < 0.05) in the B-MOS groups compared to the B-GLY groups and the control groups. The *Pseudomonas* spp. count significantly decreased (*p* < 0.05) in the tilapia intestinal samples reared in B-MOS compared to B-GLY in comparison with the control groups.

### 3.6. The Effect of Different Dietary Protein Sources and Carbon Sources on Nile Tilapia Serum Immunity Parameters 

At the end of the experimental period, all groups fed on plant protein-based diets or fish protein-based diets showed no significant differences (*p* < 0.05) in serum content of C-reactive protein, total protein and albumin, while the groups fed on fish protein-based diets showed significantly higher (*p* < 0.05) serum content of IgM and higher serum lysozyme activity than groups fed on plant protein-based diets in contrast with the serum globulin content (Table 7). In terms of the main effect of the carbon source, the B-MOS reared groups recorded significantly higher (*p* < 0.05) serum values of IgM, lysozymes activity, total protein, albumin and globulin compared to the B-GLY reared groups, while both recorded higher serum immune parameters than control groups. The C-reactive protein was lower in B-MOS reared groups than B-GLY reared groups and control groups.

The inflammatory C-reactive protein level in fish serum decreased in response to the interaction of carbon source and protein source, and for B-MOS groups either fed on a plant or fish protein, this difference was significant compared to the other experimental groups. Lysozymes activity, IgM total protein and globulin showed significantly increased (*p* < 0.05) values in the B-MOS fish protein group compared to the B-MOS plant protein group, B-GLY plant protein group and B-GLY fish protein group compared to the control groups, while albumin was significantly (*p* < 0.05) increased in both the B-MOS and B-GLY fish protein groups compared to the other groups.

### 3.7. Effect of Different Dietary Protein Sources and Carbon Sources on Nile Tilapia Transcriptomic Profile of Proinflammatory Cytokines (IL-8 and IFN-γ) Prechallenge

**Figure 2:**
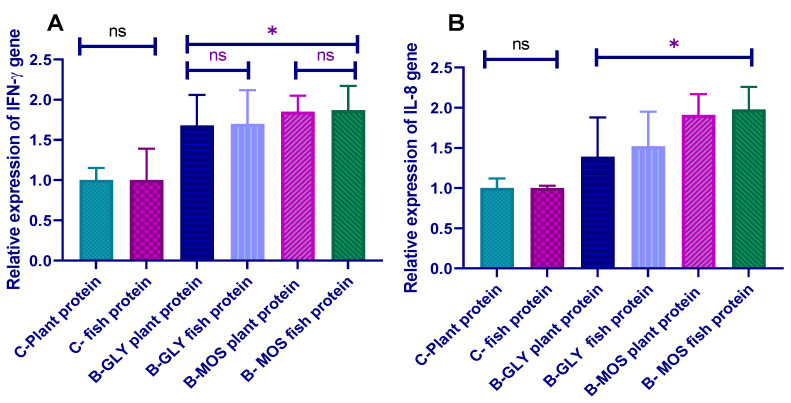
Effect of dietary protein source and carbon source on proinflammatory cytokine (**A**) IFN-γ: interferon gamma and (**B**) IL-8: interleukin-8) expression profile in Nile tilapia prechallenge (data are expressed as mean ± SEM). * Bars with an asterisk correspond to values that are significantly different (Tukey, *p* < 0.05); ^ns^ Bars with (ns) correspond to values that are not significantly different.

The mRNA expression profile of IL-8 and IFN-γ encoding genes was significantly upregulated in groups reared on the biofloc system (Figure 2). The expression of selected proinflammatory cytokines was markedly upregulated in the B-MOS groups compared to the B-GLY groups when each one was compared with the respective control groups. Moreover, the expression of IL-8 and IFN-γ was upregulated in the B-MOS groups regardless of the protein source.

### 3.8. Challenge Test with Aeromonas hydrophila and Survival Percent

**Figure 3:**
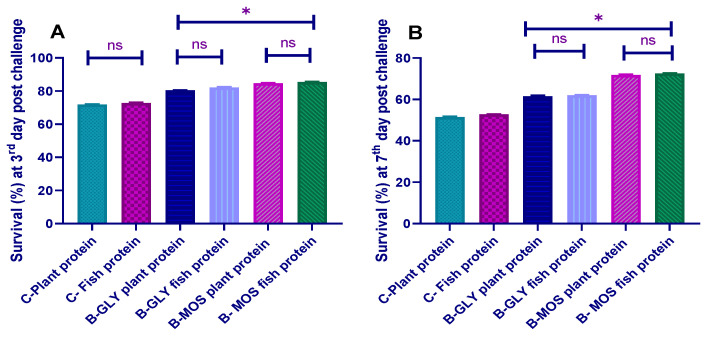
Effect of dietary protein source and carbon source on survival percent at 3 (**A**) and 7 days (**B**) postchallenge in Nile tilapia (data are expressed as mean ± SEM). * Bars with an asterisk correspond to values that are significantly different (Tukey, *p* < 0.05) ns bars, with ns corresponding to values that are not significantly different.

The clinically challenged fish of the control groups manifested clinical signs such as loss of scales, severe erythematic skin, ascites, fin rot and hemorrhage at the caudal peduncle region (Figure 3). The B-GLY reared groups manifested with a moderate erythematic skin, red ulcers, fin rot, and skin hemorrhage, and the B-MOS groups manifested with mild fin rot and mild hemorrhage at caudal peduncle with fishes with otherwise healthy appearances. In general, the severity of symptoms after *A. hydrophila* challenge was much lower in fish reared in B-MOS than in other groups.

#### 3.8.1. The Cumulative Survival Percent

The highest cumulative survival percentage of the fish after three days of challenge test was recorded in the B-MOS reared groups either fed a plant or fish protein source (84.6% and 85.4%), respectively, followed by the B-GLY groups either fed a plant or fish protein source (80.4% and 82.2%) compared to their respective control groups (71.8% and 72.2%). Seven days postchallenge, the cumulative survival percent was decreased in the B-MOS reared groups (71.8% and 72.5%), followed by the B-GLY reared groups (61.5% and 62.0%), when compared to their respective control groups (51.4% and 52.7%), regardless of the source of dietary protein.

#### 3.8.2. *Aeromonas hydrophila* Real-Time PCR Quantification and Aerolysin Virulence Gene 

**Figure 4:**
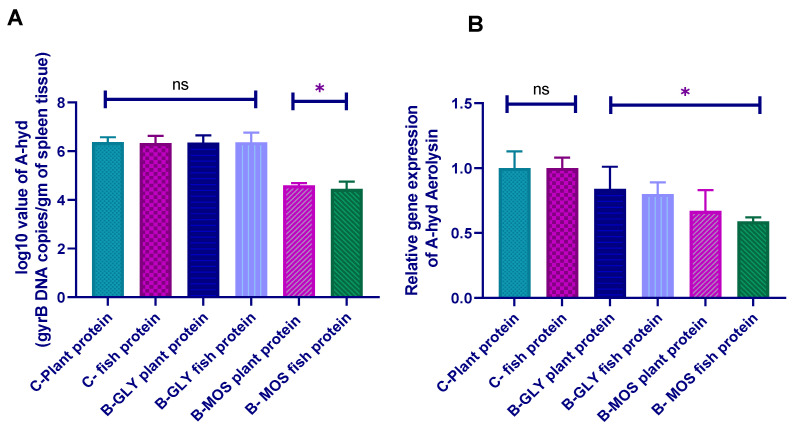
Effect of dietary protein source and carbon source on (**A**): *Aeromonas hydrophila* gry-B gene measured by quantitative real time PCR results (log 10 copies / gm spleen tissue), *(**B**): *Aeromonas hydrophila* Aerolysin virulence gene expression (data are expressed as mean ± SEM). * Bars with an asterisk correspond to values that are significantly different (Tukey, *p* < 0.05) ns bars, with ns corresponding to values that are not significantly different.

Before the challenge experiment, spleen tissues from selected fish in all experimental groups were collected and examined to ensure that they were free from *Aeromonas hydrophila* infection and all samples were free from *A. hydrophila.* The result revealed that *A. hydrophila* count (log10 CFU/gm) was decreased in the B-MOS reared groups fed on a fish and plant protein source when compared with other groups, as presented in Figure 4. Aerolysin virulence gene expression of *A. hydrophila* revealed downregulation in both B-MOS and B-GLY groups compared to the control groups, regardless of dietary protein source. 

## 4. Discussion

Using an effective carbon source in biofloc can affect the water microbial community and the nutritive value of biofloc. The addition of complex carbohydrate (prebiotics) as a carbon source in the biofloc system could enhance the nutritive value of bioflocs, which is suggested to be a contributor to the better growth of fish compared to glycerol [12]. Nile tilapia was recognized as a fish that could consume bacteria directly from the water column or bacteria that are attached to the substrate; so, its intestinal bacterial community will be affected accordingly by water bacterial contents [54]. With a limited fish protein source, microbial flocs may act as a supplementary protein source, especially when using an alternative plant protein source. In the present study, we have shown that modulating the bacterial population in the biofloc system by using mannanoligosaccharides (MOS) as a prebiotic carbon source with an alternative plant protein source can beneficially affect the water quality and productive performance of Nile tilapia.

Using an MOS biofloc system with different protein sources maintained water quality parameters including temperature, dissolved oxygen and salinity within the optimal range throughout the experiment, as evidenced by the well-developed biofloc system with improved water quality [55]. Widanarni et al. [56] reported small fluctuations in water temperature and dissolved oxygen in biofloc but within the normal range. Water pH tended to be more acidic in B-MOS groups compared with B-GLY groups than the control group; the acidic pH of water in the biofloc system may be due to photosynthesis and nitrification processes that affect carbon dioxide concentrations in water and affect water buffering capacity. Additionally, the respiration processes of microorganisms on the biofloc community influence the water pH [57]. Decreasing pH could be related to the ammonia assimilation by heterotrophic bacteria, a process that increases the CO_2_ concentration and lowers both the alkalinity and the water pH [55]. Generally, toxic concentrations of NO_2_ and NO_3_ for aquaculture are higher than 5 and 60 mg L^−1^, respectively, in the case of no water chloramination by Cl_2_: N at a ratio of 6:1, which oxidize the nitrite and nitrate in water [58]. In our study, the levels of TAN, NO_2_ and NO_3_ decreased compared with the controls and fluctuated within the normal range; this is consistent with the findings of [59]. Total ammonia nitrogen (TAN), nitrite N and nitrate N were significantly lowered in the biofloc system regardless of the diet protein source. Chen et al. [60] reported lower water pH due to the chemolithotrophic nitrification process which resulted from CaCO3 utilization and the liberation of CO_2_ and H+ into the culture medium. Inclusion of MOS in biofloc also caused the diversion of bacteria by increasing lactic acid bacteria proliferation, which assimilates MOS and secretes lactic acid in water, thus contributing to the acidic pH of water [61]. Thilakan et al. [62] also reported that lowered TAN may be due to the uptake of nitrogen during microbial proliferation. Avnimelech et al. [23] achieved improvements in water quality by using a high ratio of carbon/nitrogen by including a suitable carbohydrate source, as MOS increases the conversion of inorganic nitrogen in water to microbial nitrogen. On the other hand, the biofloc volume was significantly increased in biofloc reared groups. The increase in biofloc volume over time was due to increased total suspended solids and floc microorganism community proliferation [63]. This result is inconsistent with [64], who reported that the biofloc system reduced water alkalinity, TAN, nitrite and nitrate nitrogen, as the water nitrogen from fish excretion and feed waste was recycled and reused in the microbial formation which was engulfed by fish. Additionally, MOS inclusion increases lactic acid bacterial proliferation, which reduces water alkalinity and pH accordingly [62]. 

The best growth performance parameters were observed when using MOS as a carbon source in the biofloc fish rearing system (B-MOS), either with plant or fish protein source; this was followed by the biofloc glycerol-based carbon source (B-GLY) reared system. The lowest performance parameters were recorded for control groups. Thus, our data concluded that the fish protein source can be replaced by the plant protein source in the fish feed when MOS is added to the biofloc rearing system with a positive effect on performance parameters. Using fish meal in tilapia feed is most commonly used to meet all of the amino acid requirements of the fish but has a high cost. Biofloc technology in intensive tilapia culture offers a microbial source of protein for nourishing fish through nitrogen waste recycling to produce biofloc that is consumed by fish [65]. A proximate chemical analysis of biofloc comprised about 25% crude protein (CP), 0.6% lipids, 26% carbohydrates and 47% ash [66]; thus, nutrient content of biofloc can improve fish growth performance and weight gain and improves feed conversion [67,68], which agreed with our finding, as FCR in our study was improved below 0.9 in biofloc reared fish. The improvements in growth performance including weight gain, specific growth rate, protein efficiency ratio and reduced feed conversion ratio agree with the findings of [69], who explained that the cause is the continuous availability of food as biofloc and its high nutrient content as 27% CP and about 4.6% lipid [68]. In this study, the better growth performance of Nile tilapia reared under the biofloc system and fed on a plant or fish protein source can be attributed to the addition of MOS as an external carbon source, which caused heterotrophic bacteria to assimilate the inorganic nitrogen by adjusting the carbon-to-nitrogen ratio of water, thus resulting in the higher production of microbial protein for fish and an improvement in water quality [5,66]. Moreover, the addition of MOS as the carbon source in biofloc leads to the formation of biofloc which is a supplemental protein source for Nile tilapia, especially in groups fed on plant protein. Increasing the growth of bacteria and other microbial organisms in biofloc systems supplemented with MOS can lead to the aggregation of suspended biofloc particles in the water, and these can be eaten by omnivorous fish [4]. Biofloc is also a rich source of essential amino acids and an effective recycling process for feed nutrients [70]. Mannan oligosaccharides (MOS) not only act as a carbon source but are considered a prebiotic carbohydrate, reported to improve growth performance by increasing the proliferation of lactic acid bacteria in the intestine of fish [71]. Lactic acid bacteria can secrete mannanase enzyme which digests MOS and produces fermentation acids such as lactic acid and citric acid [16,17]. Thus, the inclusion of MOS as a carbon source in the biofloc system increased lactic acid bacteria proliferation and the presence of both MOS and lactic acid bacteria (symbiotic effect) in the water improved the fish growth performance and feed assimilation; these findings are in accordance with [72].

The improvement in nutrient digestibility in the biofloc system may be due to the secretion of extracellular enzymes by biofloc microorganisms, and these enzymes support the action of fish endogenous enzymes, causing more degradation and digestion of feed nutrient [73]. Additionally, biofloc can supply Nile tilapia with external digestive enzymes [74,75], activating indigenous digestive enzymes of reared fish (cellulase, amylase, lipase, protease of reared animals) [76,77]. The increased nutrient digestibility recorded by [68] in the biofloc system can positively affect the secretion of fish endogenous digestive enzymes (protease and amylase). This study’s results are in agreement with those reported by [78], who reported increased growth performance and feed utilization improvement by the addition of MOS to adult sea bream diets. Our results are consistent with the findings of [79], who reported that nutrient digestibility in salmon fish was improved by supplementing with MOS, which can be explained by the increased intestinal absorption surface and improved integrity of the epithelial wall lining of intestinal villi. The improved performance, feed assimilation and nutrient digestibility of Nile tilapia in our study may be related to the high number of lactic acid bacteria which can be transmitted from water to the intestines of fish and, consequently, act as probiotic bacteria [80]. Furthermore, the synergism between MOS and lactic acid bacteria in B-MOS creates an ideal gut condition, resulting in higher nutrient digestion and absorption. Additionally, [81] reported that the same synergism can improve gut morphology by increasing intestinal villus height and crypt depth. The improved water quality and production of biofloc particles due to MOS detected in our study may be attributed to both the degradability of its components and to the particle size formed, which would have increased the surface area for bacterial growth, resulting in an increased biofloc volume. This view is supported by Ferreira et al. [82], who found that increasing the substrate surface area in biofloc tanks improved water quality and increased food availability.

The biofloc system depends on the proliferation of bacteria in water. All bacterial species counts increased gradually through the experimental period. However, control groups exhibited no significant change in *Bacillus* spp., *Vibrio* spp., *Aeromonas* spp. and *Pseudomonas* spp., as the water of these tanks was changed continuously. Additionally, the inclusion of MOS as a carbon source in the biofloc system mostly affected Bacillus spp. In accordance with our findings, [83] described that most species of bacteria in the biofloc system increased in number by increasing the rearing period—the more carbon added to adjust the carbon/nitrogen ratio, the more bacterial proliferation can occur [83]; these proliferated biofloc bacteria can consume ammonia waste in water and lower the total ammonia concentration. The addition of MOS as a carbon source in the biofloc system may increase the diversity in the bacterial population: *Lactobacillus* spp. secreting mannanase enzymes enable it to digest mannan oligosaccharide [61], so *Bacillus* spp. become more abundant in biofloc with an MOS carbon source. In biofloc cultures, nonpathogenic bacteria also proliferate, which are considered to have possible probiotic potential [84,85]. The *Vibrio* spp. count increased linearly with the increased period of fish culture in the biofloc system. The count of heterophilic bacteria, characterized by the efficient utilization of various types of carbohydrates, increased with the increased addition of carbon sources to the biofloc culture [86]. Our finding was in accordance with that of Pérez-Fuentes et al. [87], who reported the isolation of more than 20 bacterial species from a tilapia culture in a biofloc system including *Bacillus* spp., *Vibrio* spp., *Aeromonas* spp. and *Pseudomonas* spp. and *Micrococcus* spp. They also reported that the load of bacteria in culture water was greater than that isolated from intestinal content. The water bacterial community in the biofloc system was not affected by protein source in fish ration. 

The biofloc system was able to generate high concentrations of bacteria which influence the digestive tract bacteria bacterial load of Nile tilapia, specifically those of the intestine. Bacteria in the biofloc culture medium affect shrimp intestines by modifying bacterial biota and replacing the pathogenic bacteria, thus improving the health of the shrimps [83]. In accordance with our results, the total *Bacillus* spp. count was increased in the B-MOS groups compared to other groups, while counts of *Vibrio* spp., *Aeromonas* spp. and *Pseudomonas* spp. decreased in fish intestinal content. Feeding prebiotics such as MOS to fish improved gut health and intestinal microbiota diversity [88]. The same observation was reported by Nedaei et al. [89], as the supplementation of prebiotics in crayfish feed increased intestinal lactic acid bacteria. There was an interaction between the inclusion of MOS in the biofloc system and increased lactic acid bacteria proliferation in water and, fish intestine (acting as probiotic bacteria), which suppress pathogenic microfloral populations through the synthesis of antimicrobial compounds, competing with pathogenic microorganisms on nutrients and decreasing adhesion of pathogenic microorganisms to the fish intestinal wall [90]. However, the bacterial population in fish intestinal content was not affected by protein source.

The immune system has plenty of mechanisms responsible for the protection against invading antigens, through humoral and cell-mediated pathways. The production of compounds, such as total protein, albumin, immunoglobulins, serum lysozymes, proteins of the acute phase inflammation as C- reactive protein and cytokines by the host could be used as markers for evaluating the innate immune response against invading pathogens.

The inflammatory C-reactive protein level in fish serum decreased in response to the interaction of the carbon source and protein source. This decrease was significant for the B-MOS groups either fed on a plant or fish protein. Lysozyme activity, IgM total protein and globulin showed gradually higher values in the B-MOS fish protein group than the B-MOS plant protein group, B-GLY plant protein group and B-GLY fish protein group, compared with their respective control groups, while albumin was increased in both B-MOS and B-GLY fish protein compared to other groups.

These results demonstrated that the biofloc system has an immunostimulatory effect on fish due to supplementing fish with a valuable nutrient that promotes immune responses. Using a fish protein source in fish meal plays an important role in maintaining the amino acid balance, which may affect the immune response of fish, as replacing fish meal with soybean meal in Atlantic salmon feed resulted in a dysfunction of the intestinal barrier, and chronic gut inflammation occurred [91]. Moreover, this harmful effect of replacing fish meal with plant protein is greater in carnivorous fish [92]. In contrast, Promthale et al. [93] explained that the inclusion of biofloc in shrimp nutrition can replace fish meal by up to 100% without any adverse effect on performance and immune response, as biofloc contains essential amino acids and fatty acids required by fish.

The improved immune response shown in our study either in fish serum immune parameters or increased cytokines gene expression may be due to the rearing of Nile tilapia in the biofloc system enriched with MOS as a carbon source which may stimulate the growth of heterotrophic bacteria such as Lactobacillus (beneficial probiotic bacteria), and these beneficial probiotic bacteria have many benefits for fish. The cell wall of these beneficial probiotic bacteria is rich in peptidoglycans, lipopolysaccharides and b-1, 3-glucans, and these components were evidenced to have an immunostimulatory effect when engulfed by fish, as Nile tilapia is recognized as a fish that can consume bacteria directly from the water column [94]. Additionally, agreeing with our results, the inclusion of yeast cell wall extracts such as β-glucans and mannan oligosaccharides in the fish feed had a putative effect on mucosal immune proteins that protect fish against invasion intestinal pathogen. Micallef et al. [95] found that the inclusion of yeast cell wall extracts such as β-glucans and mannan oligosaccharides in the fish feed had a putative effect on mucosal immune proteins that protect fish against intestinal microbial invasion. Thus, the addition of mannan oligosaccharides as a carbon source in the biofloc system increased the amount of lactic acid bacteria in water, and they had a symbiotic effect, which improved fish innate immune responses, such as increased lysozymes activity [96]. 

The mRNA expression profile of proinflammatory cytokine (IL-8 and IFN-γ) encoding genes was up-regulated in response to the biofloc system. The expression of selected proinflammatory cytokines was markedly upregulated in B-MOS groups. Our findings show that the plant protein source can be used as an alternative to fish protein source in Nile tilapia feed with a B-MOS rearing system without any adverse effect on its immunity. 

The higher fish immune response in our study may be a result of increasing the water probiotic bacterial load in the B-MOS system, which stimulates fish to secrete high molecular weight glycoproteins and promotes humoral immunity. This includes the secretion of proinflammatory cytokines, antiprotease and lysozymes for the prevention of pathogen invasion [97]. The biofloc system recycles nitrogen waste into microbial cells, increasing the water bacterial population surrounding fish and improving the immune response. Moreover, the addition of MOS to the biofloc system increased the number of natural probiotics in water reaching fish intestines and caused a greater immune response, which was reflected by the upregulation of immune genes and proinflammatory cytokines [94,98]. Additionally, MOS as a prebiotic had immune stimulatory effect on fish. 

The highest cumulative survival percent of fish was recorded in the B-MOS reared groups either fed plant or fish protein source three or seven days postchallenge. The fish resistance against challenged bacteria in the biofloc reared groups was mainly attributed to the improved immunity of these fish. Our results were in agreement with the results of Ekasari et al. [99], who described that shrimp immune response and disease resistance was improved in the biofloc system compared with control. Thus, increased fish survival percent after challenge in the B-MOS system may result from improving fish immunity and disease resistance. Additionally, the symbiotic effect of MOS and proliferated lactic acid bacteria in the fish intestine may also increase the innate immunity of fish and maximize disease resistance, increasing survival; this is in agreement with [96]. 

*Aeromonas hydrophila* DNA copies (log10 CFU/gm) decreased in the B-MOS reared groups fed on a fish and plant protein source when compared with the other groups after challenge. Aerolysin virulence gene expression of *A. hydrophila* revealed downregulation in both the B-MOS and B-GLY groups compared to the control groups, regardless of dietary protein source. 

Thus, by using the B-MOS system in Nile tilapia rearing, the count and virulence of *A. hydrophila* were decreased after challenge, while for the B-GLY system, rearing virulence of *A. hydrophila* was only decreased when compared with control groups. It has been shown that oligosaccharides such as mannan oligosaccharides (MOS) affect the immune system through mannose-binding protein secretion from the liver, which can enchain to bacteria and trigger the complementary cascade of the immune system of the host to resist the pathogen [100]. MOS is reported to increase plasma IgG and IgA and stimulate humeral immunity by increasing the secretion of the inflammatory cytokines [101]. All previously-mentioned mechanisms of disease resistance may be the main cause of the downregulation of the Aerolysin virulence gene of *Aeromonas hydrophila* after challenge and decrease in *A. hydrophila* log10 CFU count/gm in the spleen, resulting in the improved immunity, survival and disease resistance of Nile tilapia.

## 5. Conclusions

The biofloc composition, fish performance and immunity can be modulated by adding a carbon source, yet information regarding this was lacking. The result indicated that the application of a biofloc system for Nile tilapia using mannan oligosaccharides as the carbon source supported the growth of natural food (microbial flocks) and improved the water quality. This produced natural food together with dietary plant protein, enabling savings on the use of dietary fish protein and improving Nile tilapia performance and immune response. An additional benefit from using MOS in the biofloc system was modulating water and intestinal microbiota which compete with pathogens and increase disease resistance against *Aeromonas hydrophila*. In the current study, the application of MOS in the biofloc system showed positive effects on both water quality and fish performance; however, a potential limitation of its application could be the costs, as the cost benefits of using MOS as a carbon source in the biofloc system was not calculated; future studies are needed to investigate the economic viability of using MOS in a biofloc system.

## Figures and Tables

**Table 1:** Feed stuff and chemical composition of the control diets (as fed basis).

Ingredients %	Plant Protein Based Diet	Fish Protein Based Diet
corn	17	31.8
Soybean meal, (46.5% cp)	41	--
Fish meal (65% cp)	--	29
Wheat middling	7	11.6
Whole wheat flour (12.9% cp)	3.1	3.4
DDGs (28.7% cp)	4	5
Corn gluten meal (62% cp)	4.6	3.5
Rice bran	14	9
Fish oil	6.7	5.7
CaCo3	1.5	0.3
Premix *	0.5	0.5
DL- Methionine, 98%	0.4	0.2
Lysine %	0.2	--
**Calculated chemical composition ^†^**		
†DE, Kcal/Kg	2901.06	2902.91
DM %	89.06	89.45
CP, %	28.12	28.09
EE, %	11.25	11.16
CF, %	3.32	1.49
NFE %	40.22	40.64
Ash%	4.01	7.14
Ca, %	0.72	0.86
Available P, %	0.56	1.15
Lysine, %	1.62	1.70
Methionine, %	0.84	0.91
Methionine + cystine	0.90	1.07

* Vitamin and mineral mixture concentration in one kg (Vit. A 60000 I.U, D3 2.00000 I.U, E 12 gm, k3 1.2 g, Vit C. 10 g. B1 1g, B2 1.5g, B6 7.5mg, B12 6 g, biotin 5 mg, folic acid 1g, cholinHcl 15g inositol. 30 g, pantothemic acid 10 mg, nicotinic acid 10 g, paminobenzonic acid 50 mg, iron 7.5 g, copper 5 g, sodium selenite 2.5 mg, potassium iodide 1 g, zinc oxide 15 g and cobalt sulphate 450 mg). DE: digestible energy; DM: dry matter; CP: crude protein; EE: ether extract; CF: crude fiber; NFE: nitrogen free extract; Ca: calcium; P: phosphorus. ^†^ Digestible energy was calculated according to National Research Council (NRC) (1993) tables.

**Table 2:** Primer sequences used for real time PCR analysis.

Genes	Product Length (bp)	Primer Sequences (5′—>3′)	GenBank Number
**Fish genes primer sequence**			
β-actin	139	F: TGGCATCACACCTTCTATAACGA	XM_003455949.2
		R: TGGCAGGAGTGTTGAAGGTCT	
IL-8	126	F: GCA CTG CCG CTG CAT TAA G	NM_001279704.1
		R: GCA GTG GGA GTT GGG AAG AA	
IFN-γ	632	F: AGC ACA ACG TAG CTT TCC CT	XM_003460533.2
		R: TAA ACA GGG CAA ACA GGT CA	
**Bacterial Genes Primer Sequence**			
*A.* 16S	356	F: GGGAGTGCCTTCGGGAATCAGA	X74677.1
		R: TCACCGCAACATTCTGATTTG	
*A. hydrophila* (gyrB)	144	F: AGTCTGCCGCCAGTGGC	KJ747144.1
		R: CRCCCATCGCCTGTTCG	
AH-aerA	309	F: CAAGAACAAGTTCAAGTGGCCA	M16495.1
		R: ACGAAGGTGTGGTTCCAGT	

IL-8: interleukin-8; IFN-γ: interferon gamma; A-hydrophila gyrB: *Aeromonus hydrophila* DNA gyrase subunit B gene; AH-aerA: *Aeromonas hydrophila* Aerolysin virulence gene; F: Forward primer; R: Reverse primer.

**Table 3:** The effect of different dietary protein sources and carbon sources on Nile tilapia (O. niloticus) water quality parameters.

Experimental Groups	Temperature °C	pH	D. O_2_ (mg/L)	Salinity (g/L)	TAN (mg/L)	Nitrite N. (mg/L)	Nitrate N. (mg/L)	Biofloc Volume (mL)
Protein source								
Plant protein.	26.40	7.64	5.25	1.33	0.33	0.21	0.78	13.37
Fish protein.	26.40	7.62	5.27	1.33	0.32	0.22	0.77	13.48
Carbon source								
Control (C)	26.16 ^b^	8.05 ^a^	5.52 ^a^	1.32	0.47 ^a^	0.32 ^a^	1.26 ^a^	0.76 ^c^
B-GLY	26.51 ^a^	7.83 ^b^	5.19 ^b^	1.34	0.26 ^b^	0.17 ^b^	0.55 ^b^	18.57 ^b^
B-MOS	26.53 ^a^	7.01 ^c^	5.07 ^c^	1.35	0.25 ^b^	0.16 ^b^	0.52 ^b^	20.95 ^a^
Interaction protein source × carbon source								
C-plant protein	26.16 ^b^	8.07 ^a^	5.50 ^a^	1.32	0.48 ^a^	0.32 ^a^	1.27 ^a^	0.76 ^c^
C-fish protein	26.16 ^b^	8.02 ^a^	5.54 ^a^	1.31	0.46 ^a^	0.32 ^a^	1.24 ^a^	0.76 ^c^
B-GLY plant protein	26.52 ^a^	7.82 ^b^	5.18 ^b^	1.34	0.27 ^b^	0.17 ^b^	0.56 ^b^	18.62 ^b^
B-GLY fish protein	26.50 ^a^	7.83 ^b^	5.20 ^b^	1.34	0.25 ^b^	0.17 ^b^	0.53 ^b^	18.52 ^b^
B-MOS plant protein	26.52 ^a^	7.02 ^c^	5.07 ^c^	1.34	0.25 ^b^	0.16 ^b^	0.51 ^b^	20.72 ^a^
B-MOS fish protein	26.54 ^a^	6.99 ^c^	5.07 ^c^	1.35	0.24 ^b^	0.17 ^b^	0.52 ^b^	21.18 ^a^
*p*-value								
Protein source	1.00	0.344	0.464	1.00	0.242	0.469	0.229	0.514
Carbon source	<0.001	<0.001	<0.001	0.055	<0.001	<0.001	<0.001	<0.001
Interaction	<0.001	<0.001	<0.001	0.255	<0.001	<0.001	<0.001	<0.001
RSD	0.10	0.07	0.08	0.03	0.03	0.03	0.03	0.50

D. O_2_: dissolved oxygen_;_ TAN: Total ammonia nitrogen; Nitrite N.: Nitrite nitrogen; Nitrate N: Nitrate nitrogen; RSD: residual standard deviation. ^a–c^ Means with different superscripts in the same column are significantly different (*p* < 0.05).

**Table 4:** The effect of different dietary protein sources and carbon sources on Nile tilapia (*O. niloticus)* growth performance.

Experimental Groups	Initial BW, g/fish	Final BW, g/fish	WG, g/fish	WG, %	SGR	FI, g/fish	FCR	PER	K-factor
Protein source									
Plant protein.	7.93	49.28 ^b^	41.35	533.19	2.18	36.79	0.899	3.98	1.35
Fish protein.	8.41	50.59 ^a^	42.18	516.83	2.15	37.89	0.907	3.94	1.35
Carbon source									
Control (C)	8.63	45.56 ^c^	36.93 ^c^	436.68 ^b^	1.99 ^b^	36.99	1.01 ^a^	3.54 ^b^	1.15 ^c^
B-GLY	7.88	49.13 ^b^	41.26 ^b^	532.69 ^a,b^	2.19 ^a^	36.19	0.88 ^b^	4.03 ^a^	1.37 ^b^
B-MOS	7.99	55.10 ^a^	47.11 ^a^	605.66 ^a^	2.31 ^a^	38.83	0.82 ^b^	4.31 ^a^	1.53 ^a^
Interaction protein source × carbon source									
C-plant protein	8.27	44.24 ^d^	35.97 ^c^	438.58 ^c^	2.00 ^a,b^	36.36	1.01 ^a^	3.51 ^b^	1.11 ^c^
C-fish protein	8.98	46.88 ^c^	37.90 ^c^	434.79 ^c^	1.98 ^b^	37.62	1.00 ^a,b^	3.57 ^a,b^	1.19 ^b,c^
B-GLY plant protein	7.59	48.61 ^b,c^	41.01 ^b^	550.78 ^b^	2.22 ^a,b^	35.39	0.87 ^a,b^	4.11 ^a,b^	1.38 ^a,b^
B-GLY fish protein	8.16	49.66 ^b^	41.50 ^b^	514.59 ^b^	2.16 ^a,b^	37.00	0.89 ^a,b^	3.96 ^ab,^	1.36 ^a,b^
B-MOS plant protein	7.91	54.98 ^a^	47.07 ^a^	610.21 ^a^	2.32 ^a^	38.61	0.82 ^b^	4.33 ^a^	1.55 ^a^
B-MOS fish protein	8.08	55.22 ^a^	47.15 ^a^	601.12 ^a^	2.31 ^a,b^	39.05	0.83 ^b^	4.28 ^a^	1.51 ^a^
*p*-value									
Protein source	0.296	0.01	0.144	0.635	0.606	0.322	0.826	0.757	0.814
Carbon source	0.358	<0.001	<0.001	<0.002	<0.001	0.151	<0.001	<0.001	<0.001
Interaction	0.621	<0.001	<0.001	0.019	0.015	0.405	0.009	<0.001	<0.001
RSD	1.23	1.28	1.50	93.22	0.18	2.99	0.09	0.39	0.10

BW: body weight; WG: weight gain; SGR: specific growth rate; FI: feed intake; FCR: feed conversion ratio; PER: protein efficiency ratio; K-factor: Fulton’s condition factor (K); RSD: residual standard deviation. ^a–d^ Means with different superscripts in the same column are significantly different (*p* < 0.05).

**Table 5:** The effect of different dietary protein sources and carbon sources on Nile tilapia (*O. niloticus)* nutrient digestibility.

Experimental Groups	DM%	CP%	EE%	NFE%	Ash%
Protein source					
Plant protein.	80.19 ^b^	88.44 ^b^	93.10	72.54	35.85 ^b^
Fish protein.	81.00 ^a^	91.93 ^a^	93.19	72.54	41.99 ^a^
Carbon source					
Control (C)	78.48 ^c^	87.98 ^c^	92.35 ^b^	72.47	37.67 ^b^
B-GLY	81.05 ^b^	90.98 ^b^	93.56 ^a^	72.47	39.65 ^a^
B-MOS	82.26 ^a^	91.59 ^a^	93.52 ^a^	72.71	39.44 ^a^
Interaction protein source × carbon source					
C-plant protein	77.46 ^d^	85.45 ^e^	92.51 ^c^	72.40	34.50 ^e^
C-fish protein	79.51 ^c^	90.52 ^c^	92.20 ^c^	72.53	40.83 ^c^
B-GLY plant protein	81.01 ^b^	89.51 ^d^	93.46 ^a,b^	72.56	36.54 ^d^
B-GLY fish protein	81.09 ^b^	92.45 ^b^	93.65 ^a,b^	72.56	42.77 ^a^
B-MOS plant protein	82.11 ^a^	90.36 ^c^	93.32 ^b^	72.66	36.52 ^d^
B-MOS fish protein	82.40 ^a^	92.81 ^a^	93.72 ^a^	72.74	42.36 ^b^
*p*-value					
Protein source	<0.001	<0.001	0.354	0.973	<0.001
Carbon source	<0.001	<0.001	<0.001	0.355	<0.001
Interaction	<0.001	<0.001	<0.001	0.705	<0.001
RSD	0.44	0.26	0.27	0.42	0.28

DM: dry matter; CP: crude protein; EE: ether extract; NFE: nitrogen free extract; Ash: mineral content; RSD: residual standard deviation. ^a–e^ Means with different superscripts in the same column are significantly different (*p* < 0.05).

**Table 6:** The effect of different dietary protein sources and carbon sources on Nile tilapia (*O. niloticus)* intestinal bacterial count.

Experimental Groups	*Bacillus* spp. (cfu*10^5^/mL)	*Vibrio* spp. (cfu*10^1^/mL)	*Pseudomonas* spp. (cfu*10^3^/mL)	*Aeromonus* spp. (cfu*10^3^/mL)
Protein source				
Plant protein.	10.32	6.62	6.27	11.53
Fish protein.	10.33	6.46	6.20	11.20
Carbon source				
Control (C)	5.15 ^c^	8.49 ^a^	7.70 ^a^	13.10 ^b^
B-GLY	6.04 ^b^	8.14 ^a^	6.5 ^b^	14.00 ^a^
B-MOS	19.78 ^a^	3.00 ^b^	4.5 ^c^	7.00 ^c^
Interaction protein source x carbon source				
C- plant protein	5.17 ^c^	8.78 ^a^	7.60 ^a^	13.20 ^a^
C- fish protein	5.13 ^c^	8.79 ^a^	7.80 ^a^	13.00 ^a^
B-GLY plant protein	5.98 ^b^	8.15 ^a^	6.60 ^b^	14.20 ^a^
B-GLY fish protein	6.11 ^b^	8.15 ^a^	6.40 ^b^	13.80 ^a^
B-MOS plant protein	19.81 ^a^	2.93 ^b^	4.60 ^c^	7.20 ^b^
B-MOS fish protein	19.76 ^a^	3.06 ^b^	4.40 ^c^	6.80 ^b^
*p*-value				
Protein source	0.96	0.56	0.77	0.35
Carbon source	<0.001	<0.001	<0.001	<0.001
Interaction	<0.001	<0.001	<0.001	<0.001
RSD	0.62	0.73	0.61	0.96

CFU: colony forming unit_;_ RSD: residual standard deviation. ^a–c^ Means with different superscripts in the same column are significantly different (*p* < 0.05).

**Table 7:** The effect of different dietary protein sources and carbon sources on Nile tilapia (*O. niloticus)* serum immunity parameters.

Experimental Groups	C-Reactive Protein (mg/L)	IgM (mg/dL)	Lysozymes (U/mL)	Albumin (g/L)	Total Protein (g/L)	Globulin (g/L)
Protein source						
Plant protein.	2.98	34.78 ^b^	1.50 ^b^	1.68	3.40	1.72 ^a^
Fish protein.	3.00	35.76 ^a^	1.57 ^a^	1.92	3.61	1.69 ^b^
Carbon source						
Control (C)	3.21 ^a^	32.24 ^c^	1.11 ^c^	1.44 ^c^	2.79 ^c^	1.36 ^c^
B-GLY	3.07 ^a^	33.95 ^b^	1.65 ^b^	1.91 ^b^	3.67 ^b^	1.76 ^b^
B-MOS	2.71 ^b^	39.61 ^a^	1.85 ^a^	2.06 ^a^	4.05 ^a^	1.99 ^a^
Interaction protein source x carbon source						
C-plant protein	3.22 ^a^	31.02 ^d^	1.03 ^f^	1.27^c^	2.57 ^e^	1.30 ^c^
C-fish protein	3.20 ^a^	33.46 ^c^	1.19 ^e^	1.60 ^b^	3.02 ^d^	1.42 ^c^
B-GLY plant protein	3.06 ^a^	34.06 ^c^	1.63 ^d^	1.73 ^b^	3.63 ^c^	1.90 ^a,b^
B-GLY fish protein	3.07 ^a^	33.85 ^c^	1.66 ^c^	2.10 ^a^	3.71 ^b,c^	1.61 ^b,c^
B-MOS plant protein	2.67 ^b^	39.25 ^b^	1.83 ^b^	2.04 ^a^	3.99 ^a,b^	1.95 ^a^
B-MOS fish protein	2.74 ^b^	39.67 ^a^	1.87 ^a^	2.07 ^a^	4.12 ^a^	2.04 ^a^
*p*-value						
Protein source	0.83	<0.001	<0.001	0.028	0.770	<0.001
Carbon source	<0.001	<0.001	<0.001	<0.001	<0.001	<0.001
Interaction	0.003	<0.001	<0.001	<0.001	<0.001	<0.001
RSD	0.23	0.54	0.15	0.25	0.24	0.001

IgM: immunoglobulin-M; RSD: residual standard deviation. ^a–f^ Means with different superscripts in the same column are significantly different (*p* < 0.05).
